# Supplementary material for: Genome-Wide Association Study for Maize Hybrid Performance in a Typical Breeder Population
Source: Int J Mol Sci. 2024 Jan 18;25(2):1190. doi: 10.3390/ijms25021190 (PMC10816832; doi:10.3390/ijms25021190)
Supplement: Supplementary file 1 [file ijms-25-01190-s001.zip › ijms-2770949-supplementary/03.Supplementary figures.pdf]

Supplementary Figures

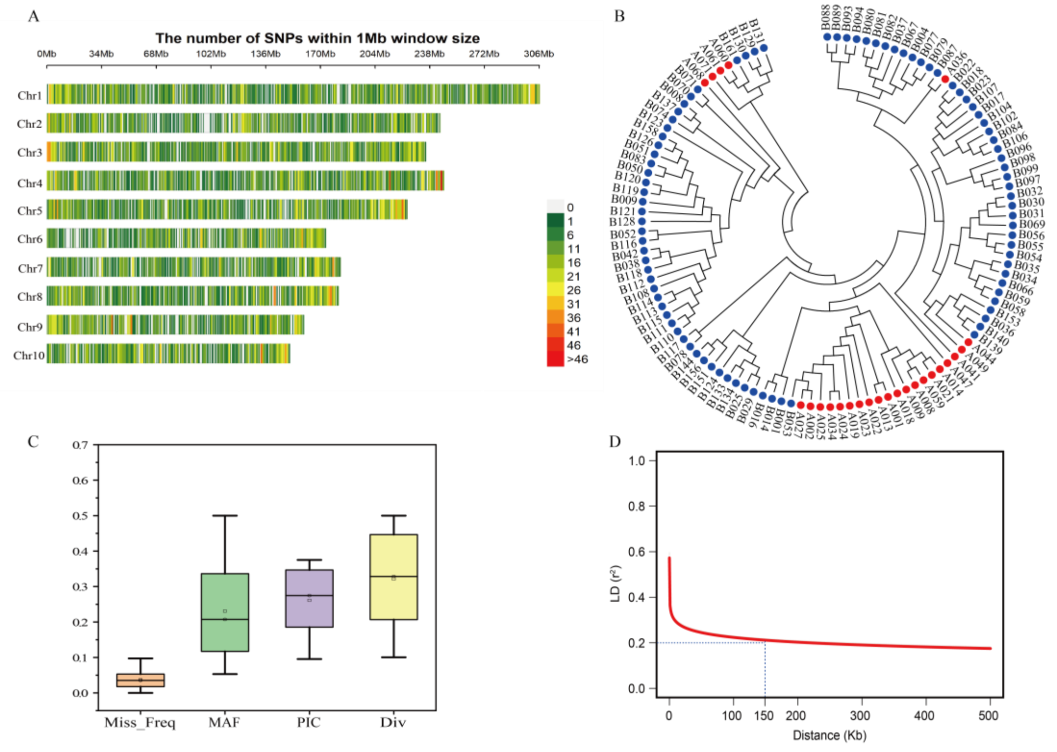

Figure S1. Genetic profile of 113 inbred lines using 19,461 SNPs. (**A**) Density distribution of SNPs across different Chromosomes; (**B**) Neighbor joining tree based on the genetic distances among 113 lines genotyped by 19,461 SNP markers.; (**C**) Descriptive statistical indicators of the 19,461 SNPs in the 113 parental materials; (**D**) Linkage disequilibrium (LD) decay in the parental population.

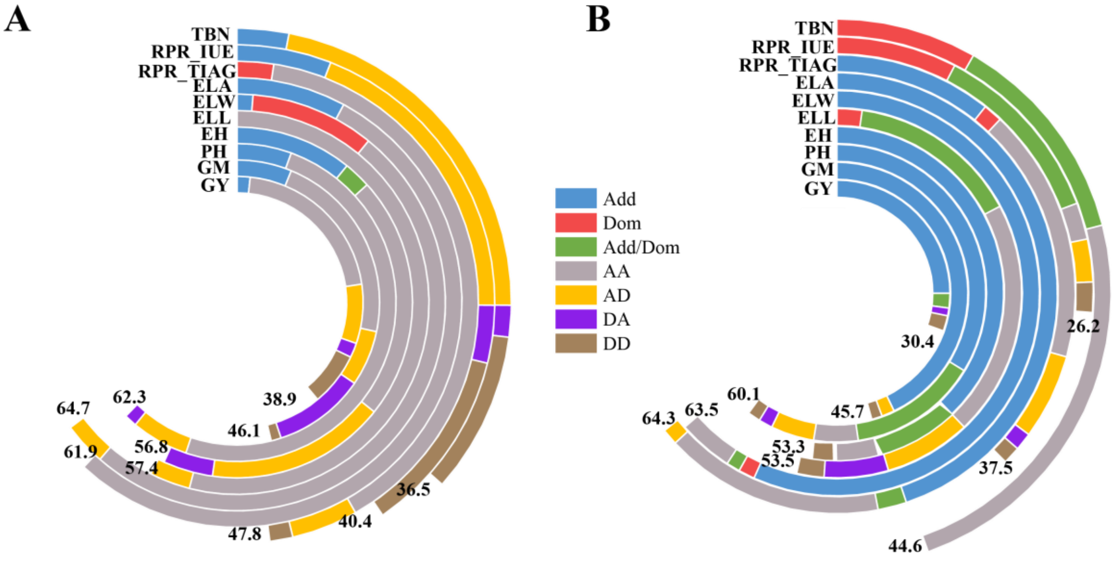

Figure S2. Phenotypic variation explained in ten traits by positive QTNs detected from models KinA (A) and KinADE (B). Add: Additive effect, Dom: dominance effect, AA: epistatic effect of Additive-by-Additive, AD: epistatic effect of Additive-by-dominance, DA: epistatic effect of dominance -by- Additive, DD: epistatic effect of dominance-by-dominance.

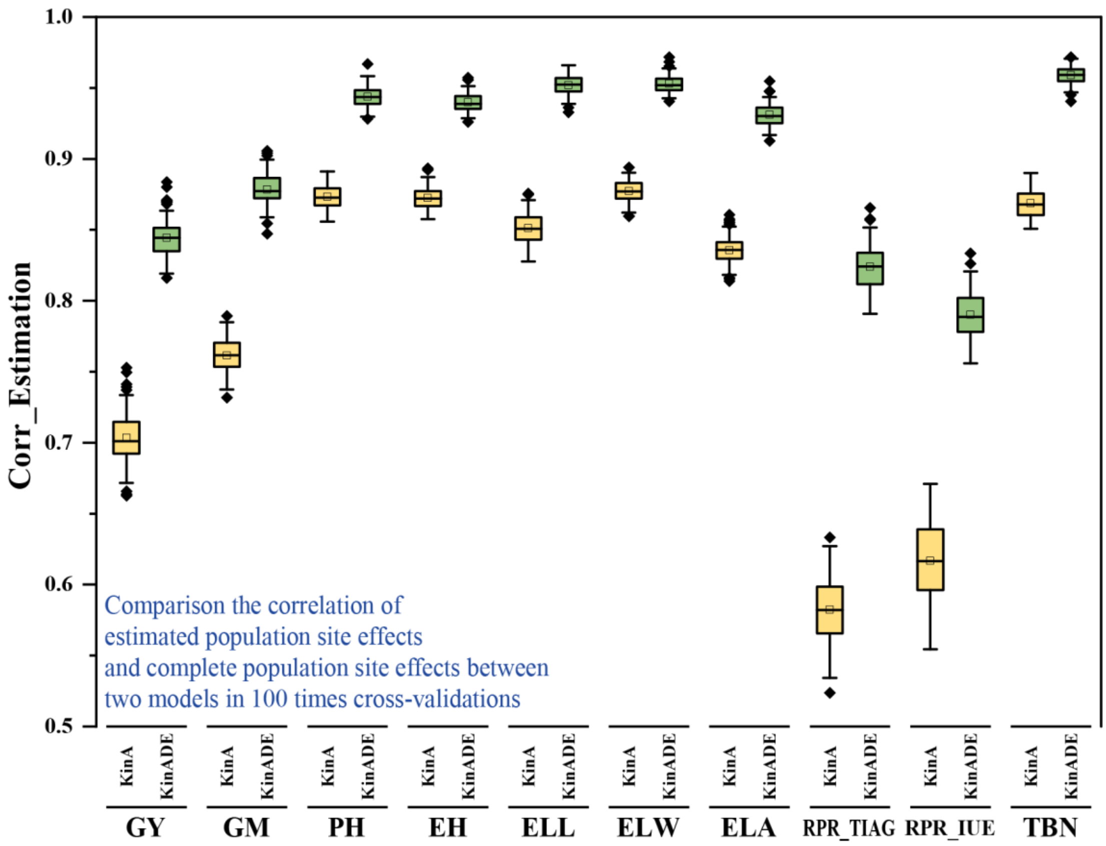

Figure S3. Comparison of estimates of the PVE for ten traits between Model KinA and KinADE based on cross validation.

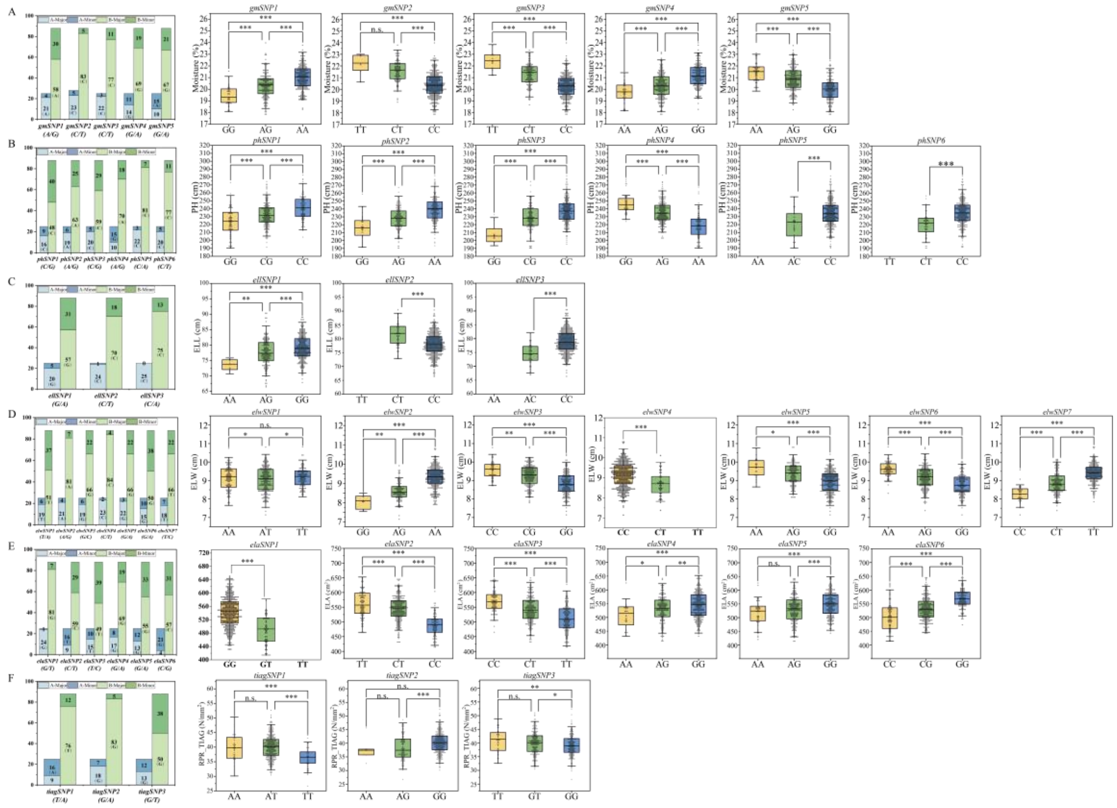

Figure S4. Allele frequency and phenotypic information of different genotypes of each main effect QTN for GM (A), PH (B), ELL (C), ELW (D), ELA (E) and RPR_TIAG (F). A-

Major and A-Minor means the globe major and globe minor allele distribution within Shaan A group; B-Major and B-Minor means the globe major and globe minor allele distribution within Shaan B group. *, **, and *** means significant at P <= 0.05, P <= 0.01, and p <= 0.001, respectively.

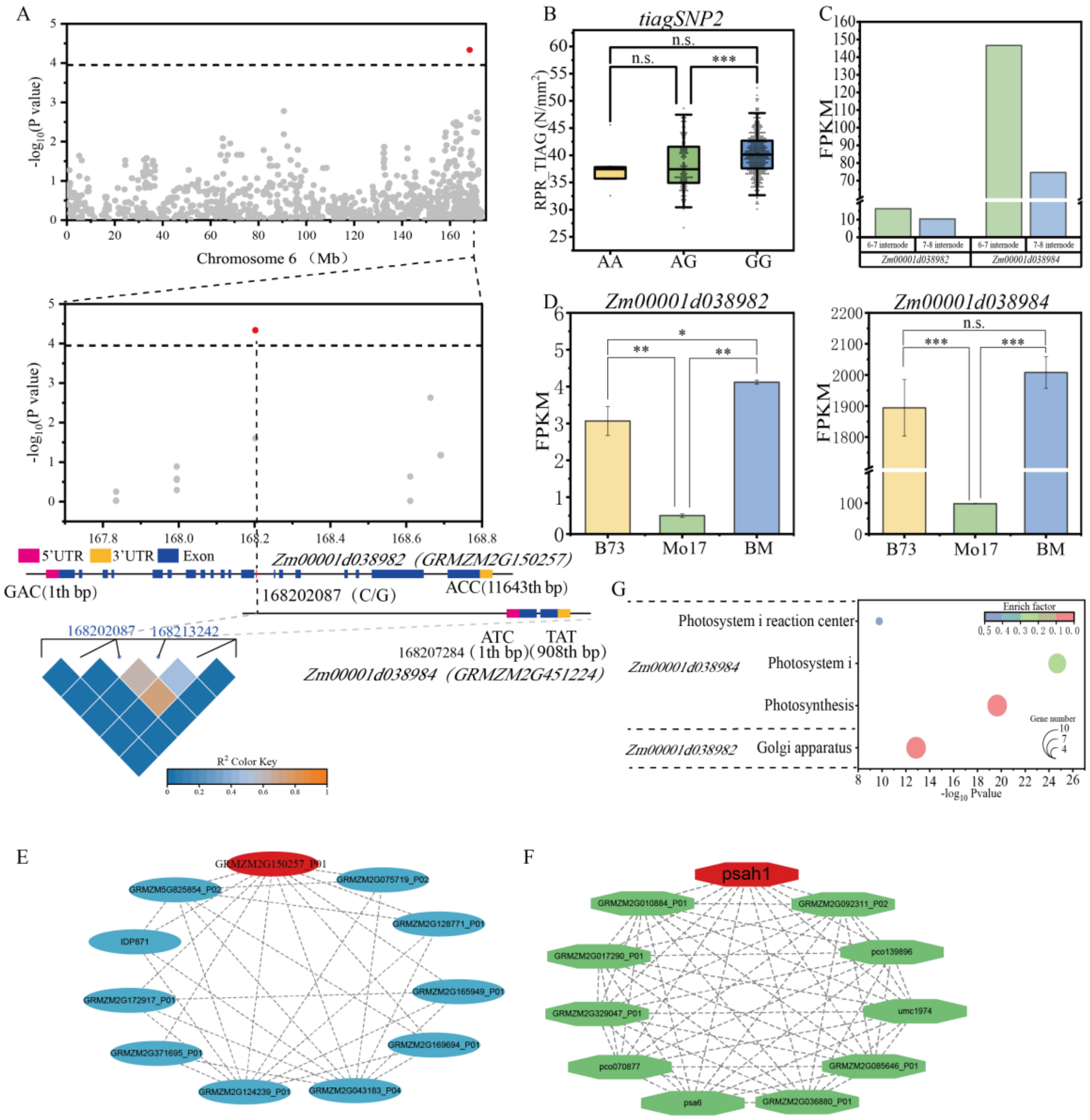

Figure S5. Multi-omic identification of candidate genes *Zm00001d038982* and *Zm00001d038984*. (**A**) Structure and mutation sites of the RPR_TIAG-related genes Zm00001d038982 and Zm00001d038984 on chromosome 6; (**B**) Phenotypic level of different genotypes in the hybrid population. ** and *** mean significance levels at 0.01 and 0.001, respectively; (**C**) Transcription level of gene *Zm00001d038982* and *Zm00001d038984* on 6-8 internode in B73; (**D**) Transcription level of gene *Zm00001d038982* and *Zm00001d038984* at seedling stage in two inbred lines and their hybrid; (**E**,**F**) Protein-protein interaction network of candidate genes *Zm00001d038982* (**E**) and *Zm00001d038984* (**F**), Red indicates the protein encoded by the candidate gene; (**G**) Biological functions of candidate genes and the interaction networks.

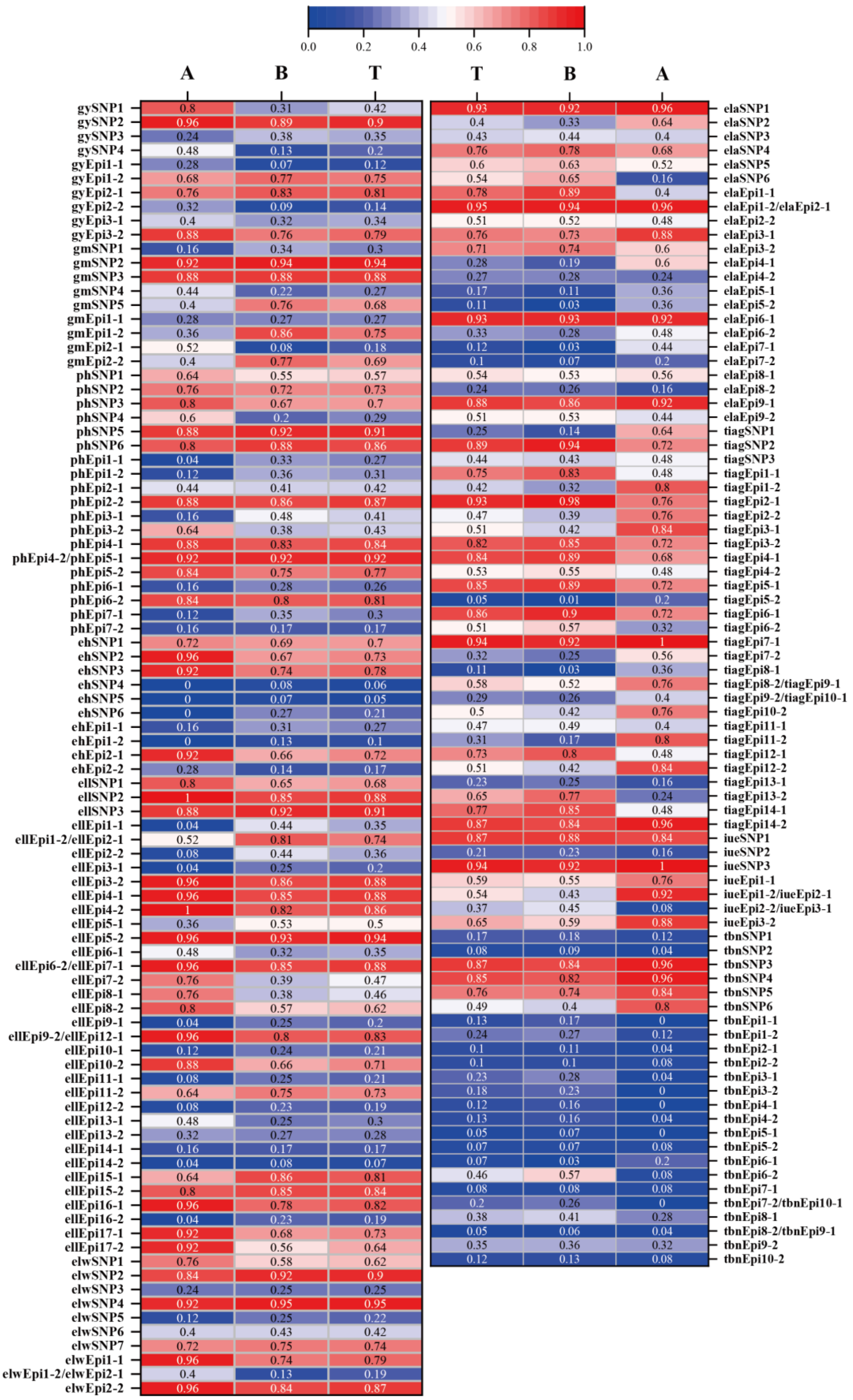

Figure S6. Enrichment degree of favorable genotypes of QTNs detected by model KinADE within ShaanA (**A**), ShaanB (**B**), and Total (T) population.

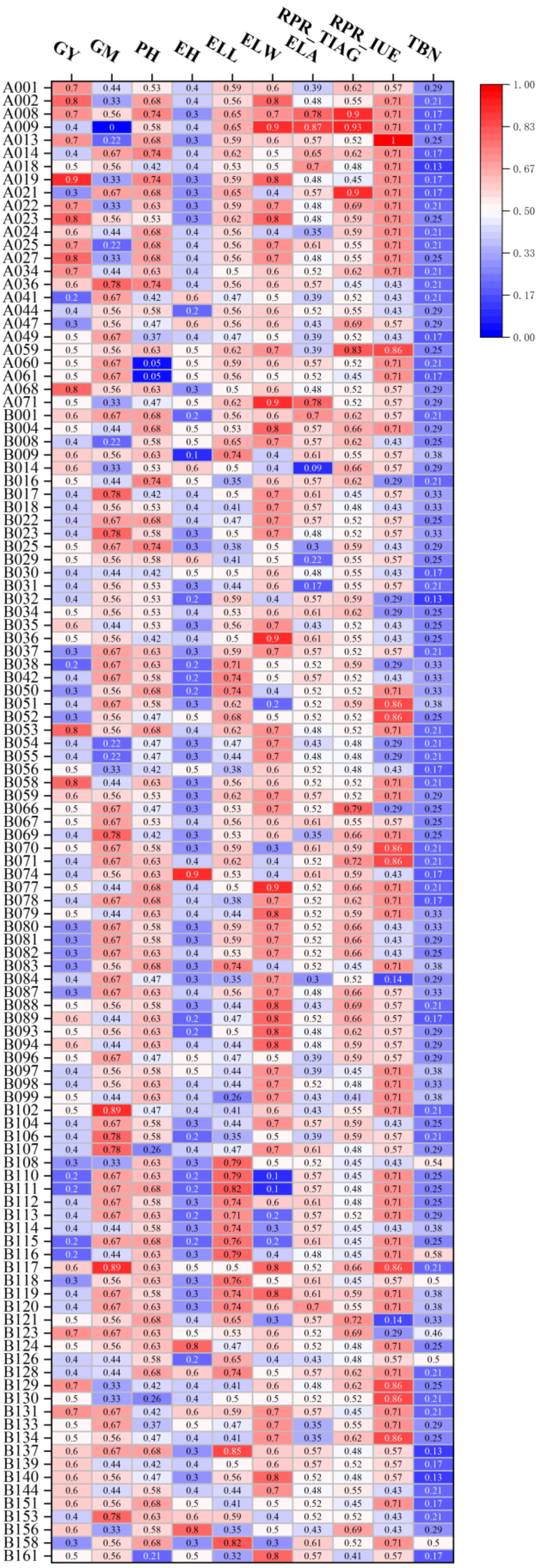

Figure S7. The enrichment degree of favorable genotypes of all QTNs in different inbred lines based on the model KinADE.
