# Supplementary material for: Genome-Wide Association Study for Maize Hybrid Performance in a Typical Breeder Population
Source: Int J Mol Sci. 2024 Jan 18;25(2):1190. doi: 10.3390/ijms25021190 (PMC10816832; doi:10.3390/ijms25021190)
Supplement: Supplementary file 1 [file ijms-25-01190-s001.zip › ijms-2770949-supplementary/04.Supplementary tables.pdf]

Supplementary Tables

Table S1. Positive epistatic QTNs identified with model KinADE.

Detailed information was available in the attached Excel file.

Table S2. Degree of dominance (d/a values) for the all QTNs identified by model KinADE.

Detailed information was available in the attached Excel file.

Table S3. Candidate genes for all QTNs identified based on the model KinADE.

Detailed information was available in the attached Excel file.

Table S4. The expression of PH, EH, RPR_TIAG and RPR_IUE candidate genes at seedling stage on B73, Mo17 and B73 × Mo17.

Detailed information was available in the attached Excel file.

Table S5. The expression of GY and GM candidate genes at spikelet stage on CL11, NG5 and HYB (CL11 × NG5).

Detailed information was available in the attached Excel file.

Table S6. The expression of GY and GM candidate genes at floret stage on CL11, NG5 and HYB (CL11 × NG5).

Detailed information was available in the attached Excel file.

Table S7. Released hybrid varieties combined from elite line A008 (KA105).

Hybrid Varieties Name	Approval No	Approved by	Female	Male
ShaanDan 679	20220397	National/China	KA105	KB179
ShaanDan 638	20220117	National/China	KA105	KB024
ShaanDan 660	20210062	National/China	KA105	KB089
ShaanDan 642	20210530	National/China	KA105	KB207
ShaanDan 620	20200264	National/China	KA105	KB106
ShaanDan 632	20220065	Province/Shaanxi	KA105	KB074
ShaanDan 697	20220063	Province/Shaanxi	KA105	KB121
ShaanDan 635	20220050	Province/Shaanxi	KA105	KB019
ShaanDan 673	20220044	Province/Shaanxi	KA105	KB066
ShaanDan 630	20220008	Province/Shaanxi	KA105	KB028
ShaanDan 608	20210055	Province/Shaanxi	KA105	KB085
ShaanDan 682	20210049	Province/Shaanxi	KA105	KB139
ShaanDan 669	2020061	Province/Shaanxi	KA105	KB109
ShaanDan 619	2020053	Province/Shaanxi	KA105	KB020
ShaanDan 662	2020046	Province/Shaanxi	KA105	KB128
ShaanDan 659	2020010	Province/Shaanxi	KA105	KB107
ShaanDan 658	2019001	Province/Shaanxi	KA105	KB025
ShaanDan 618	2015006	Province/Shaanxi	KA105	KB081
