# Supplementary figures and images for: Genome-Wide Association Study for Maize Hybrid Performance in a Typical Breeder Population
Source: Int J Mol Sci. 2024 Jan 18;25(2):1190. doi: 10.3390/ijms25021190 (PMC10816832; doi:10.3390/ijms25021190)

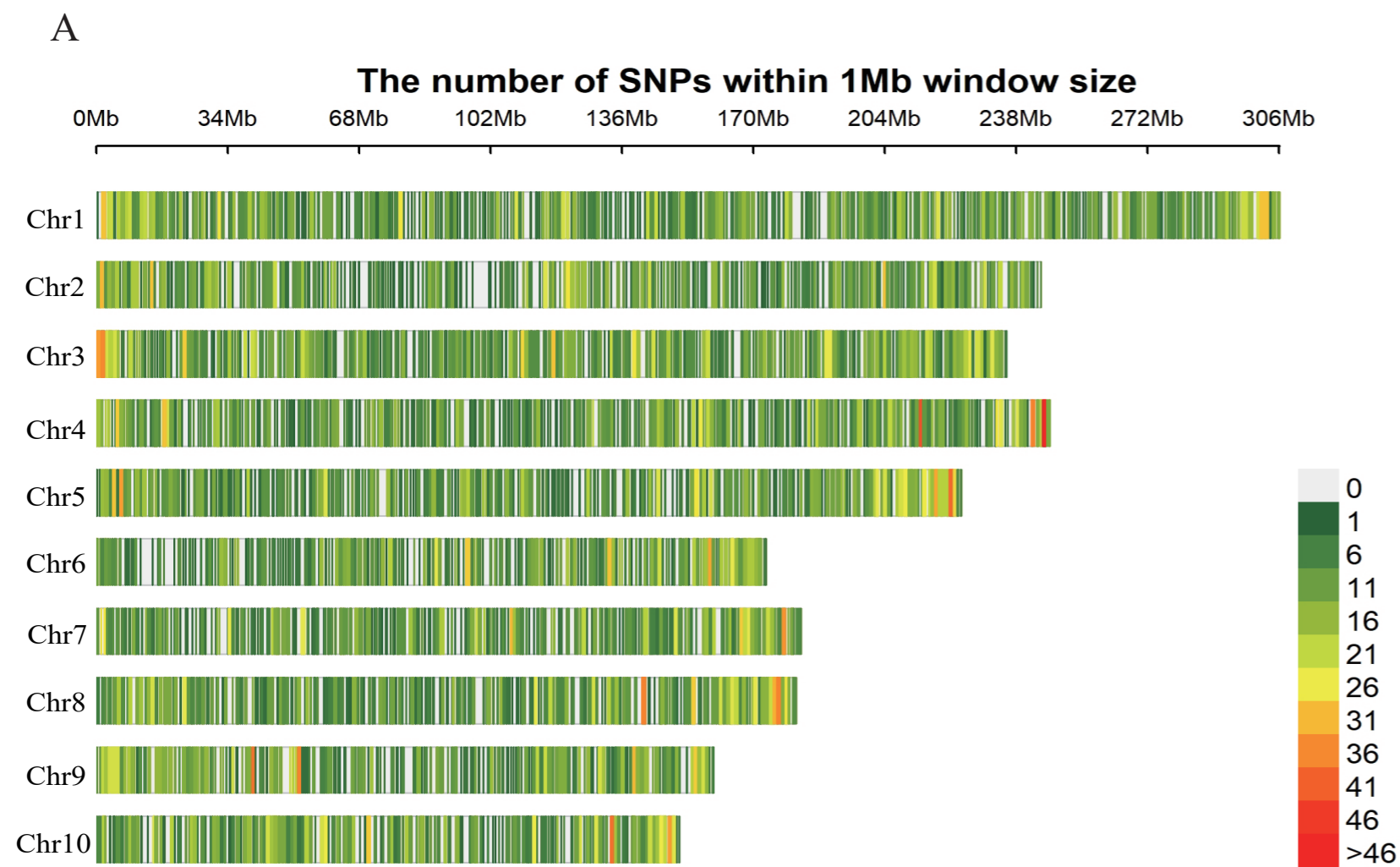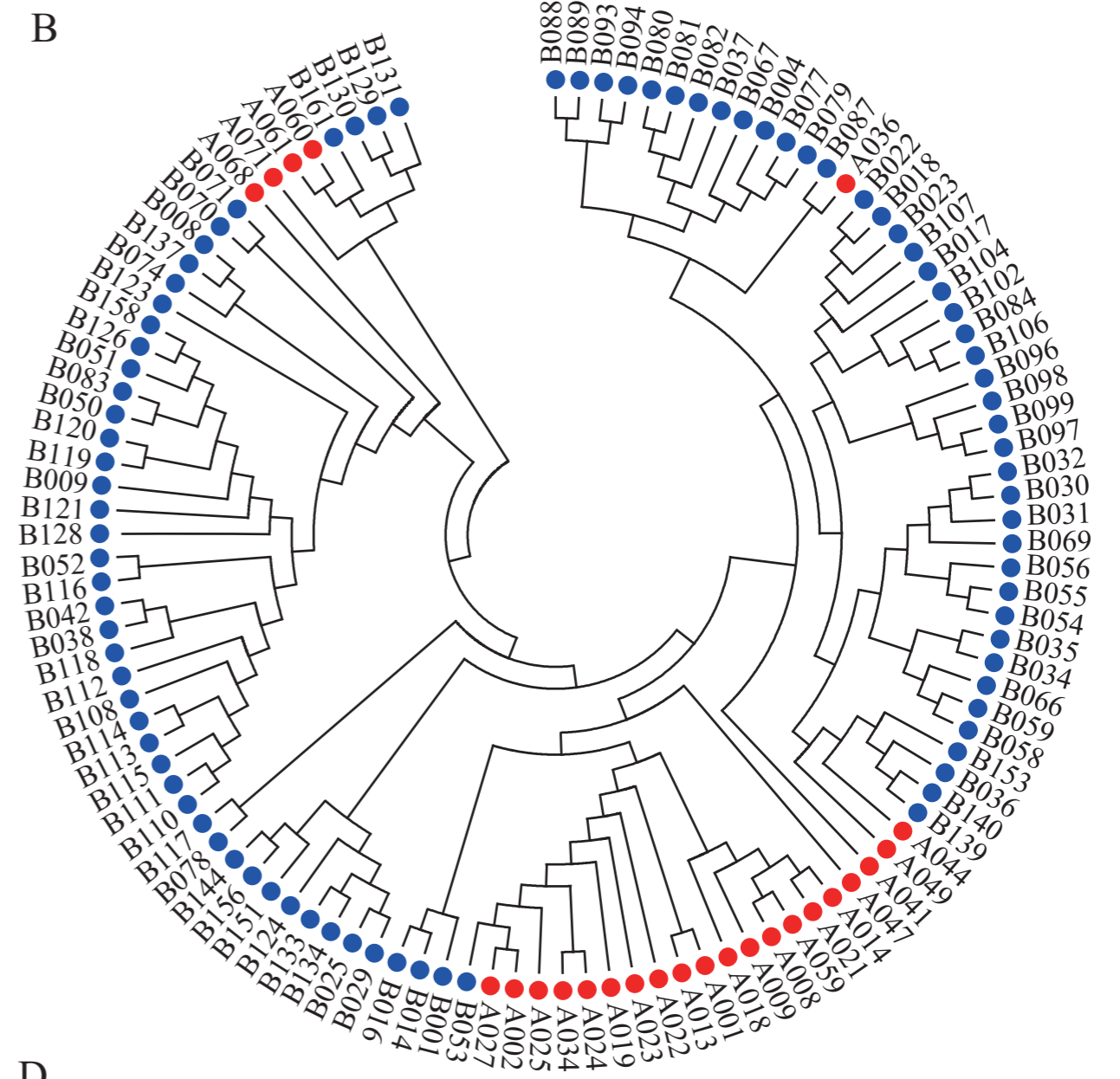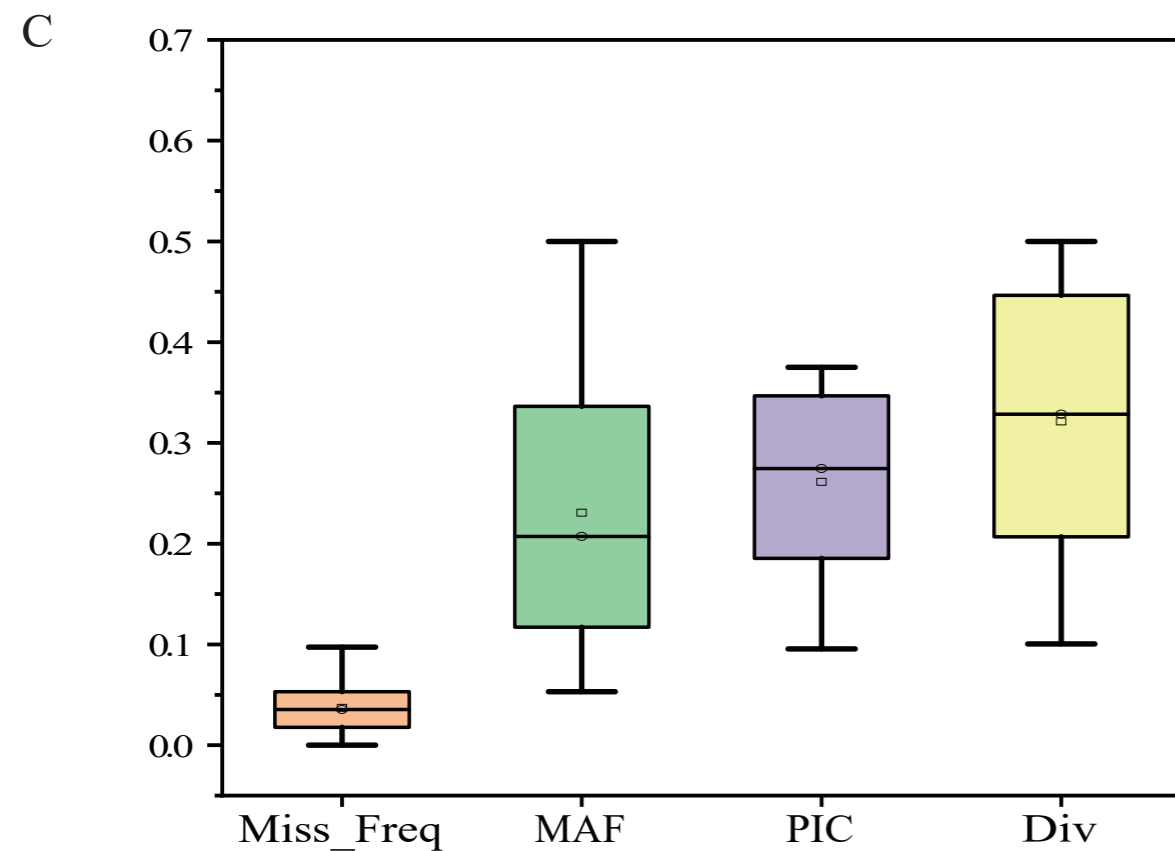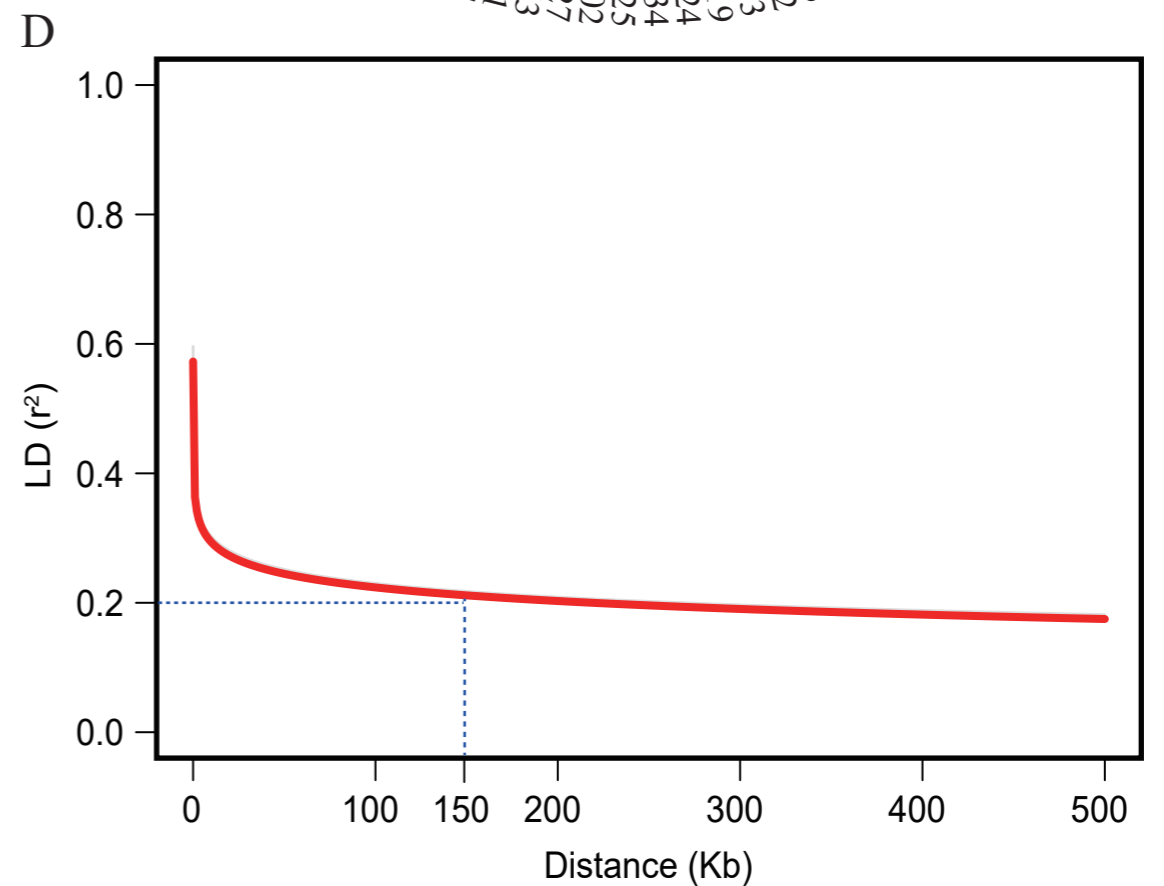

Supplement: Supplementary file 1 [file ijms-25-01190-s001.zip › ijms-2770949-supplementary/Supplementary figures/Figure S1. Genetic profile of parental lines.pdf]

A

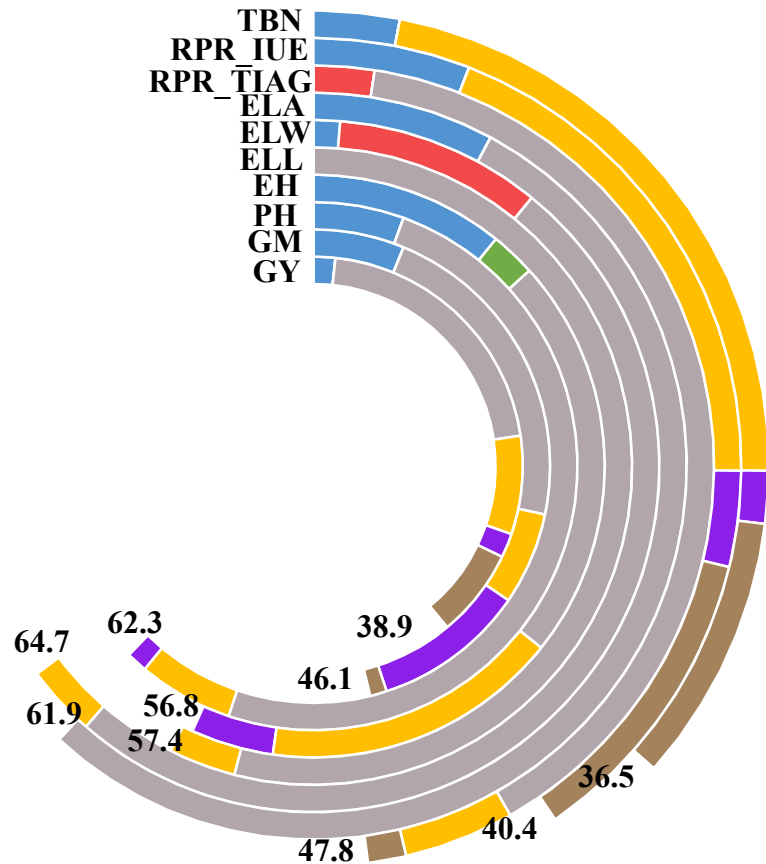

B

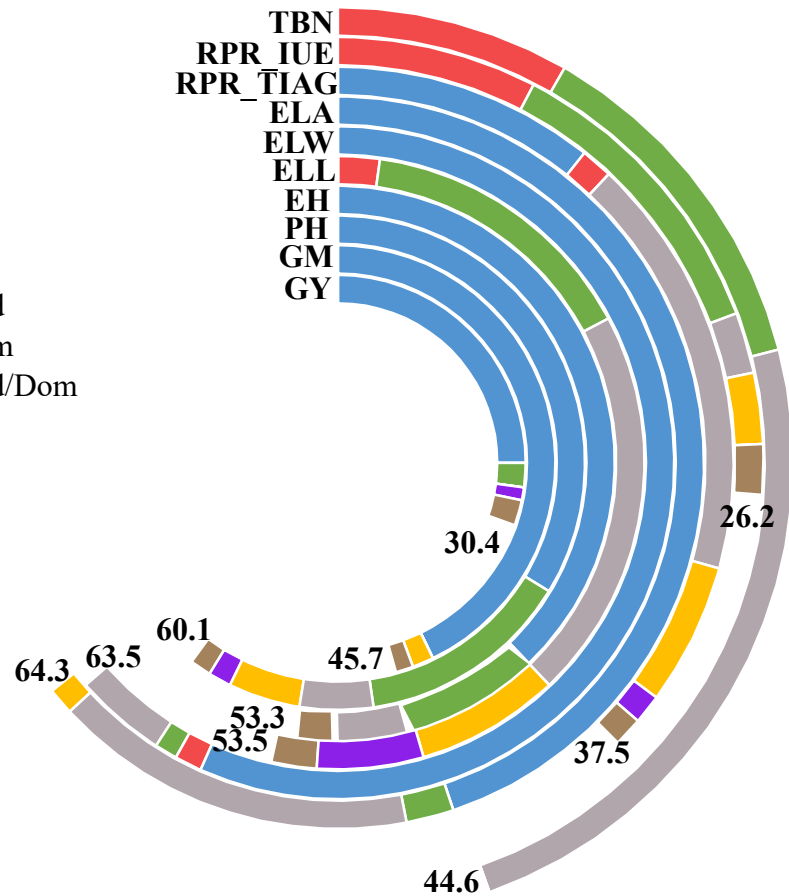

Supplement: Supplementary file 1 [file ijms-25-01190-s001.zip › ijms-2770949-supplementary/Supplementary figures/Figure S2. PVE by QTNs from both models KinA and KinADE.pdf]

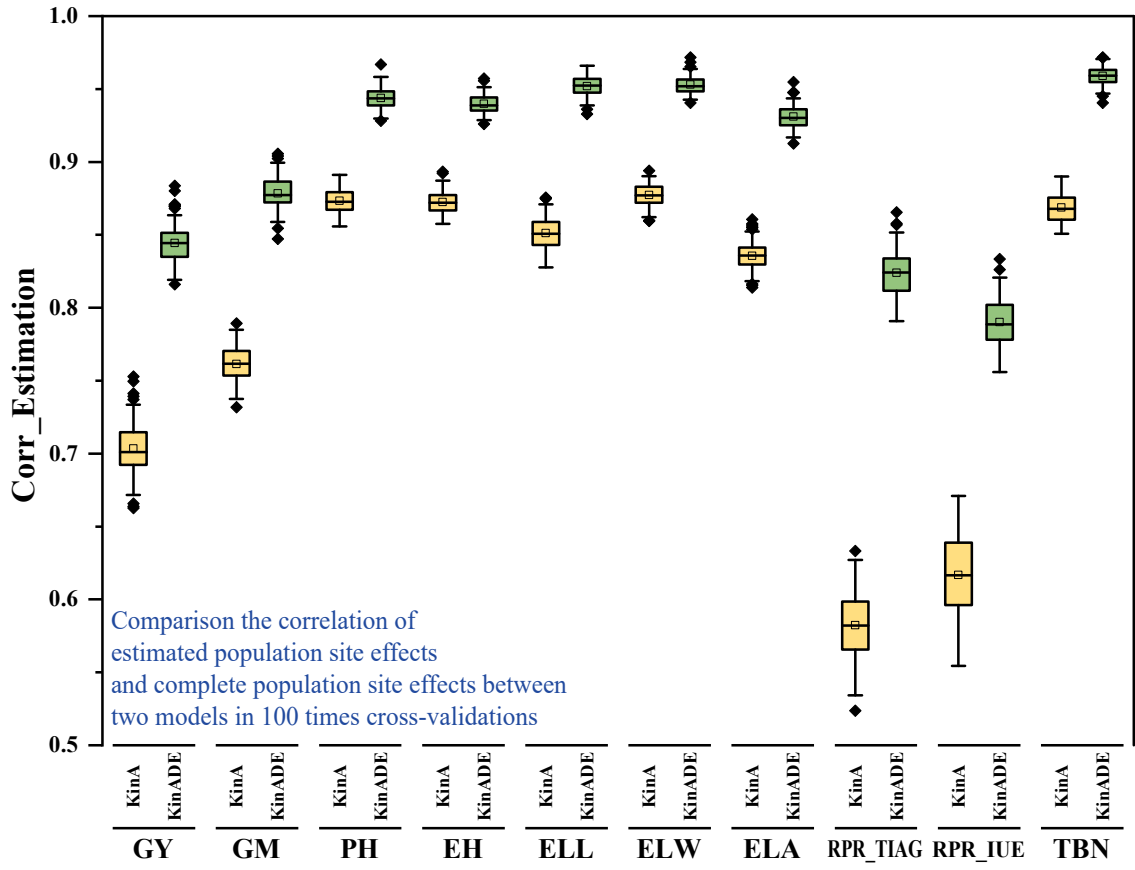

Supplement: Supplementary file 1 [file ijms-25-01190-s001.zip › ijms-2770949-supplementary/Supplementary figures/Figure S3. PVE comparison between KinA and KinADE based on cross vaildation.pdf]

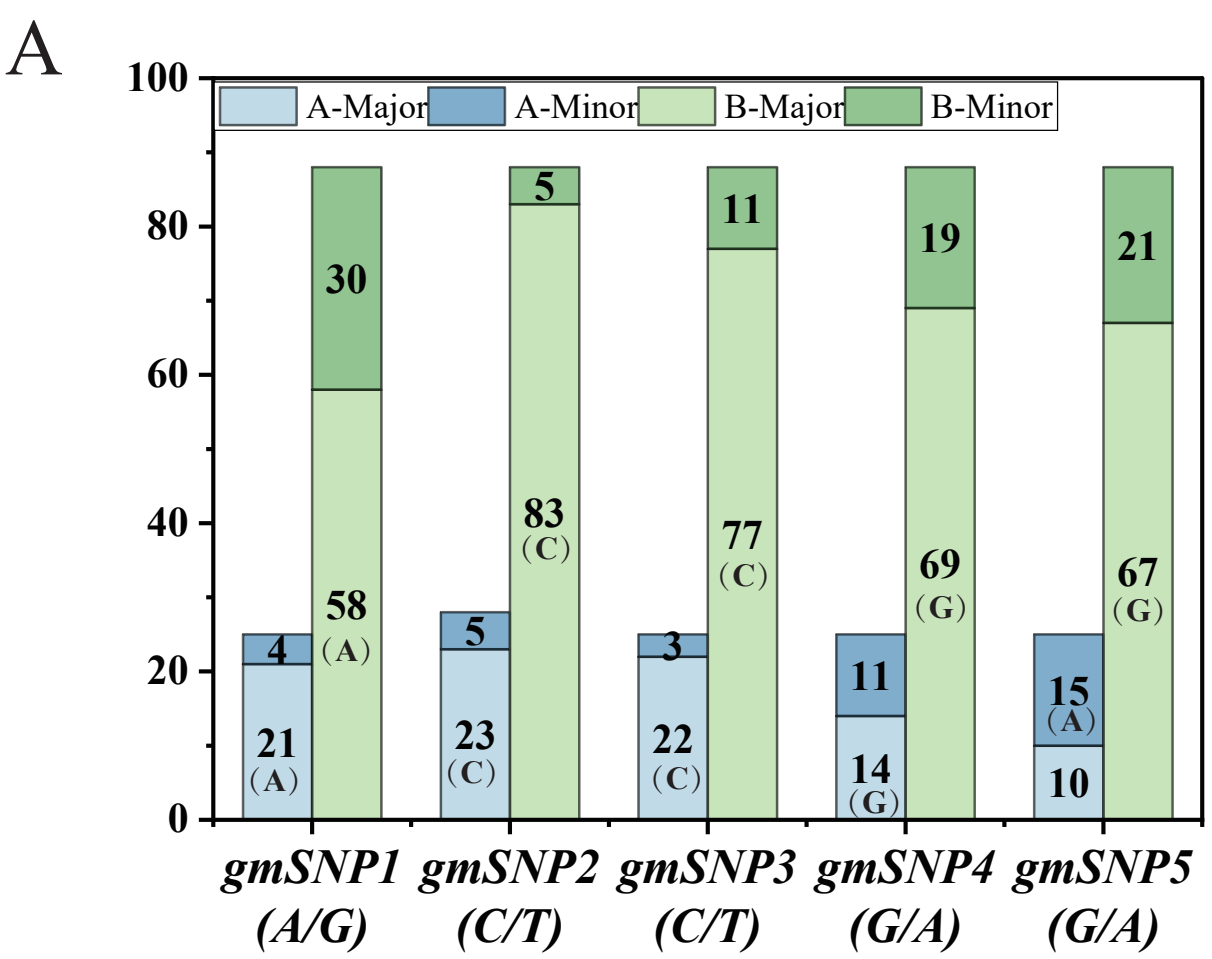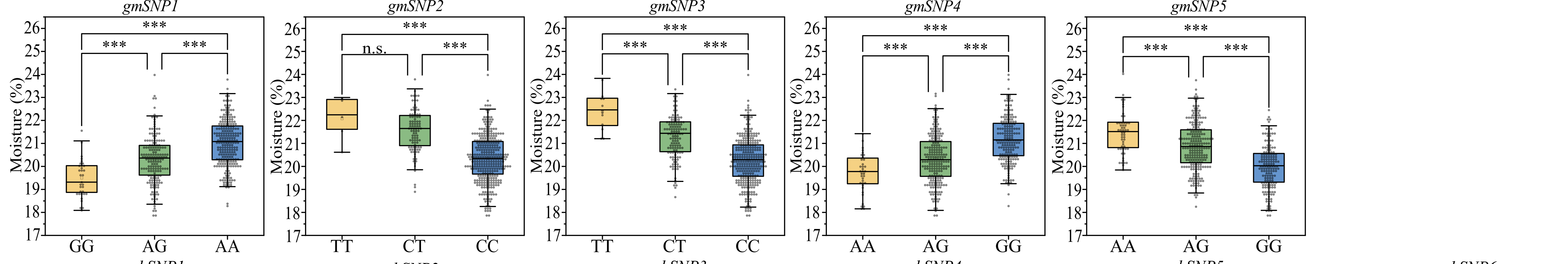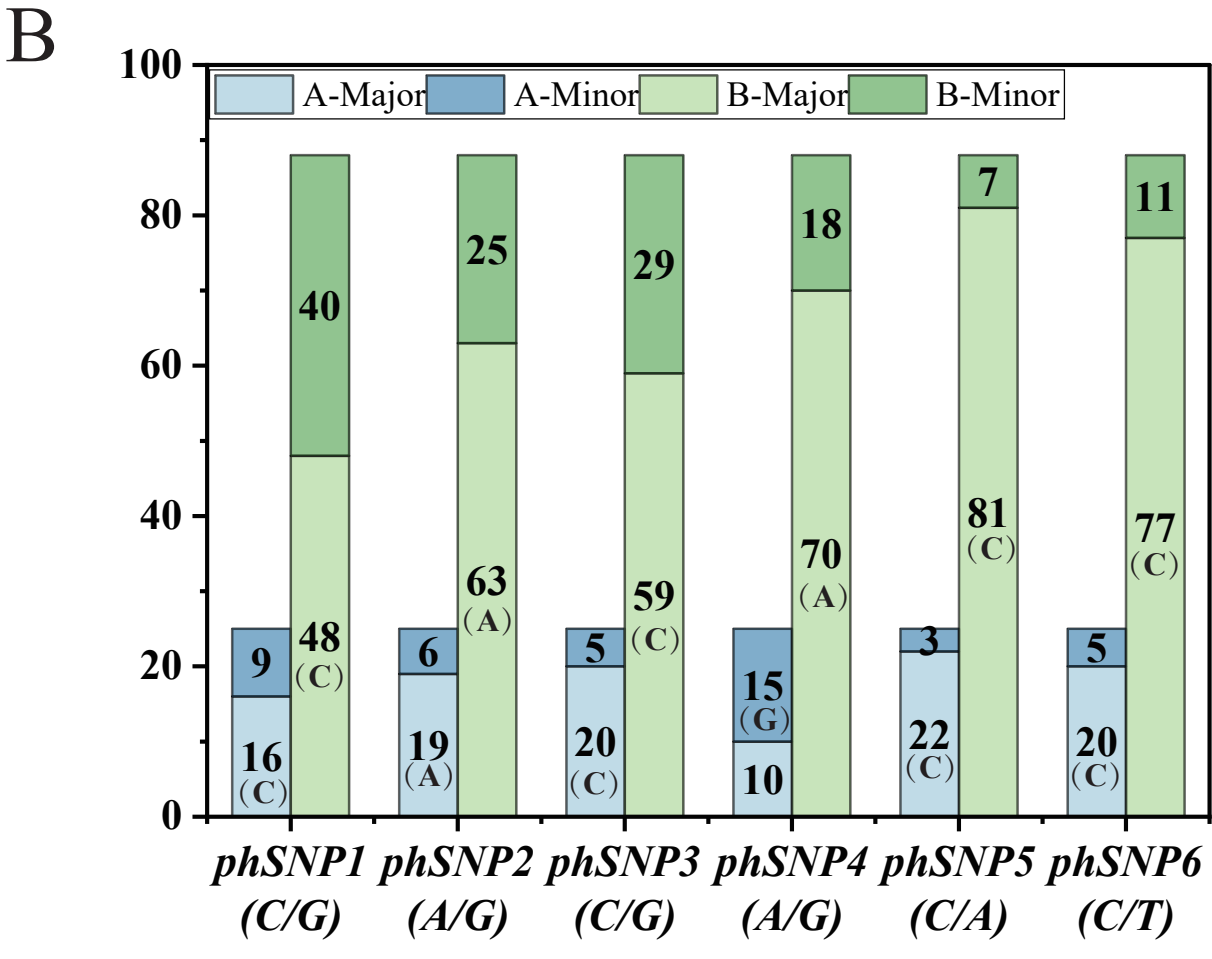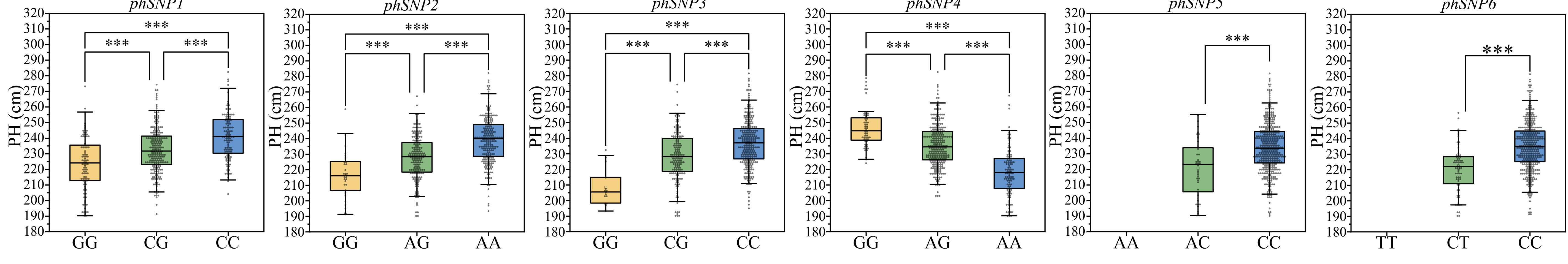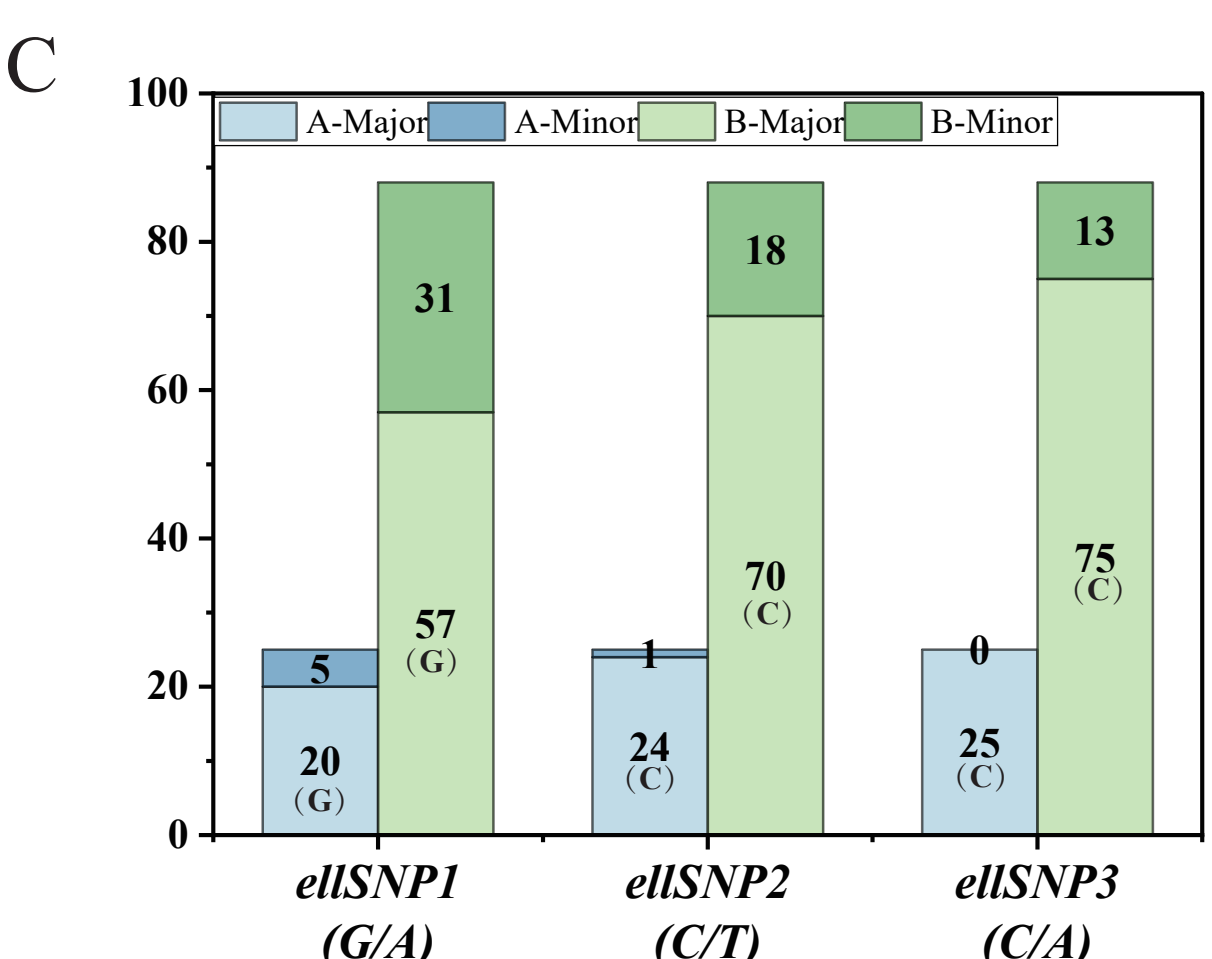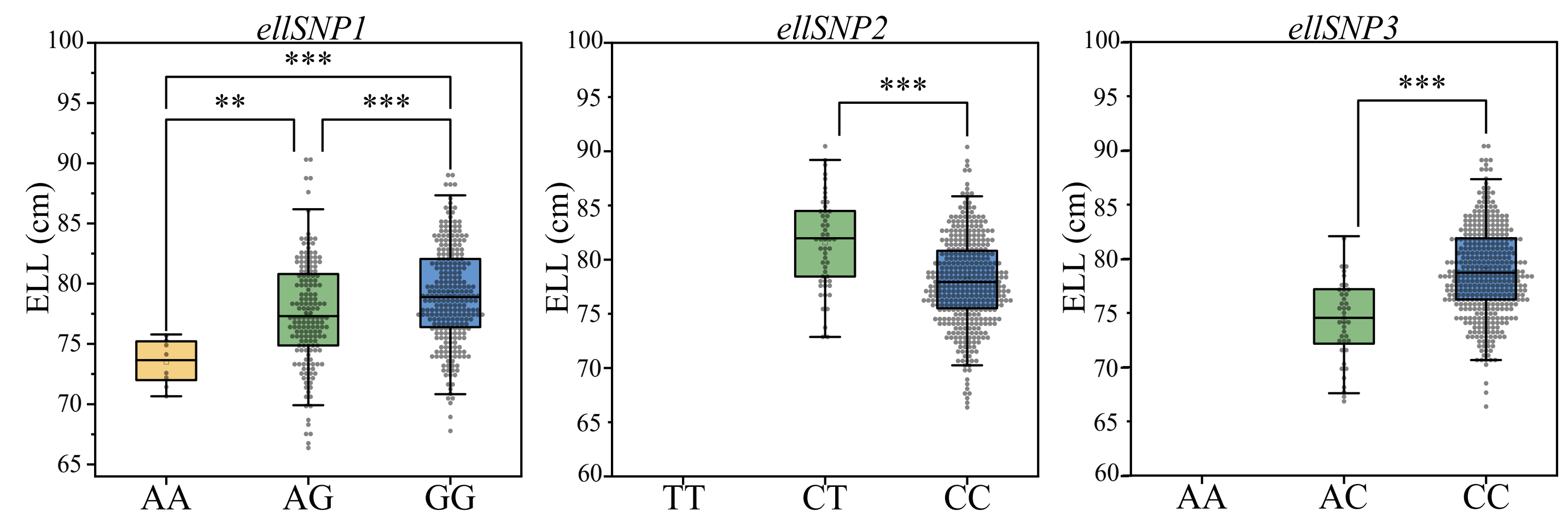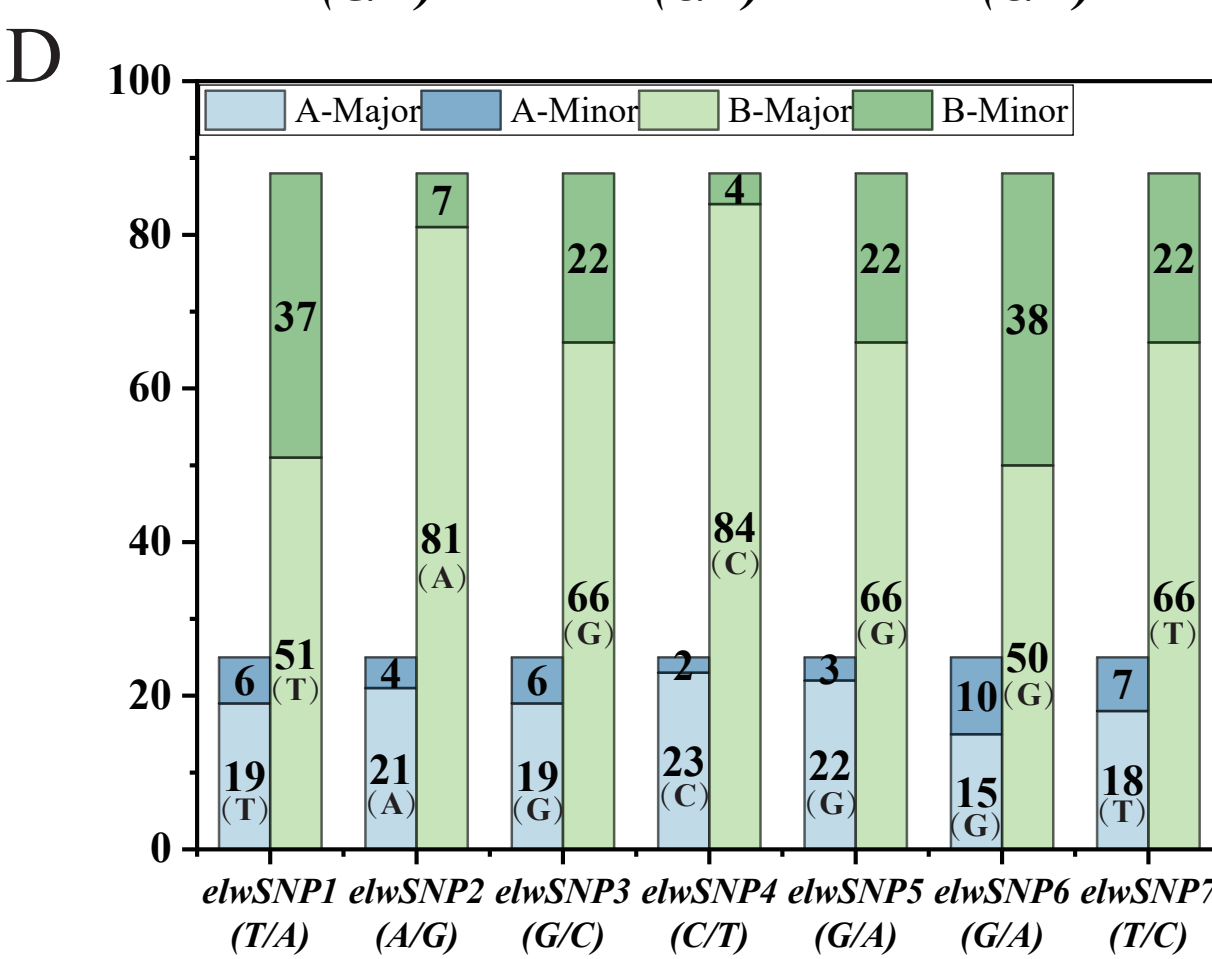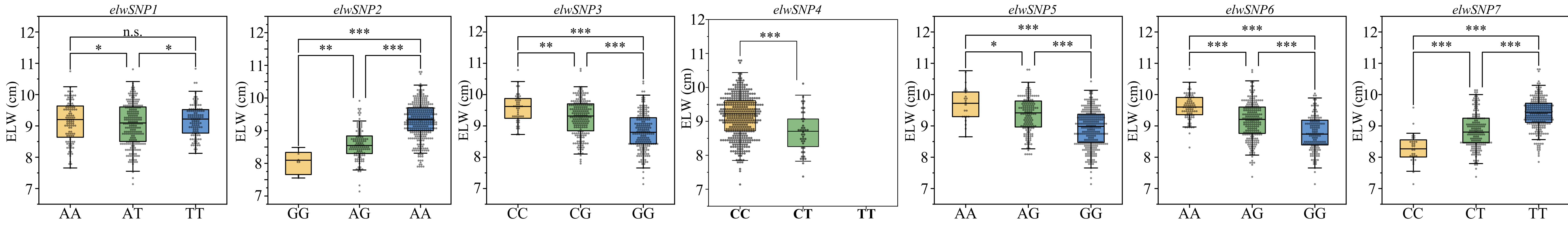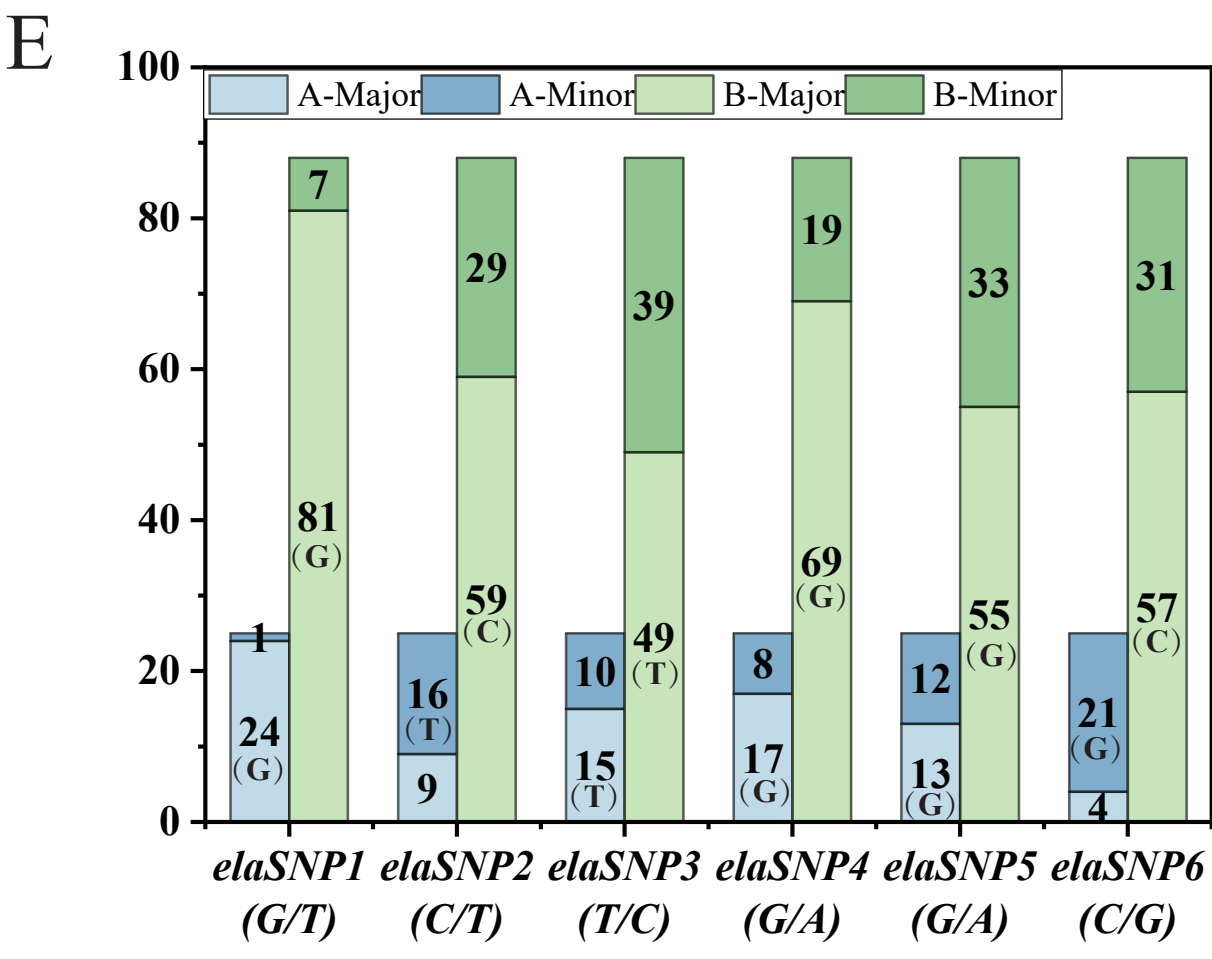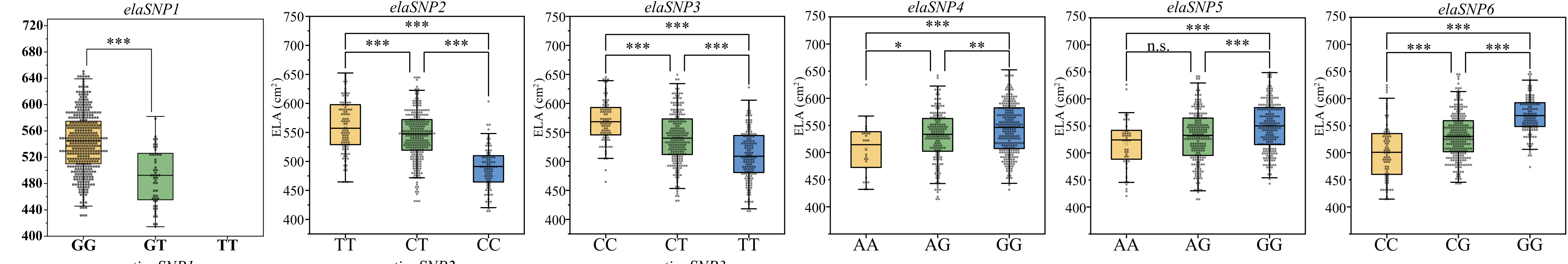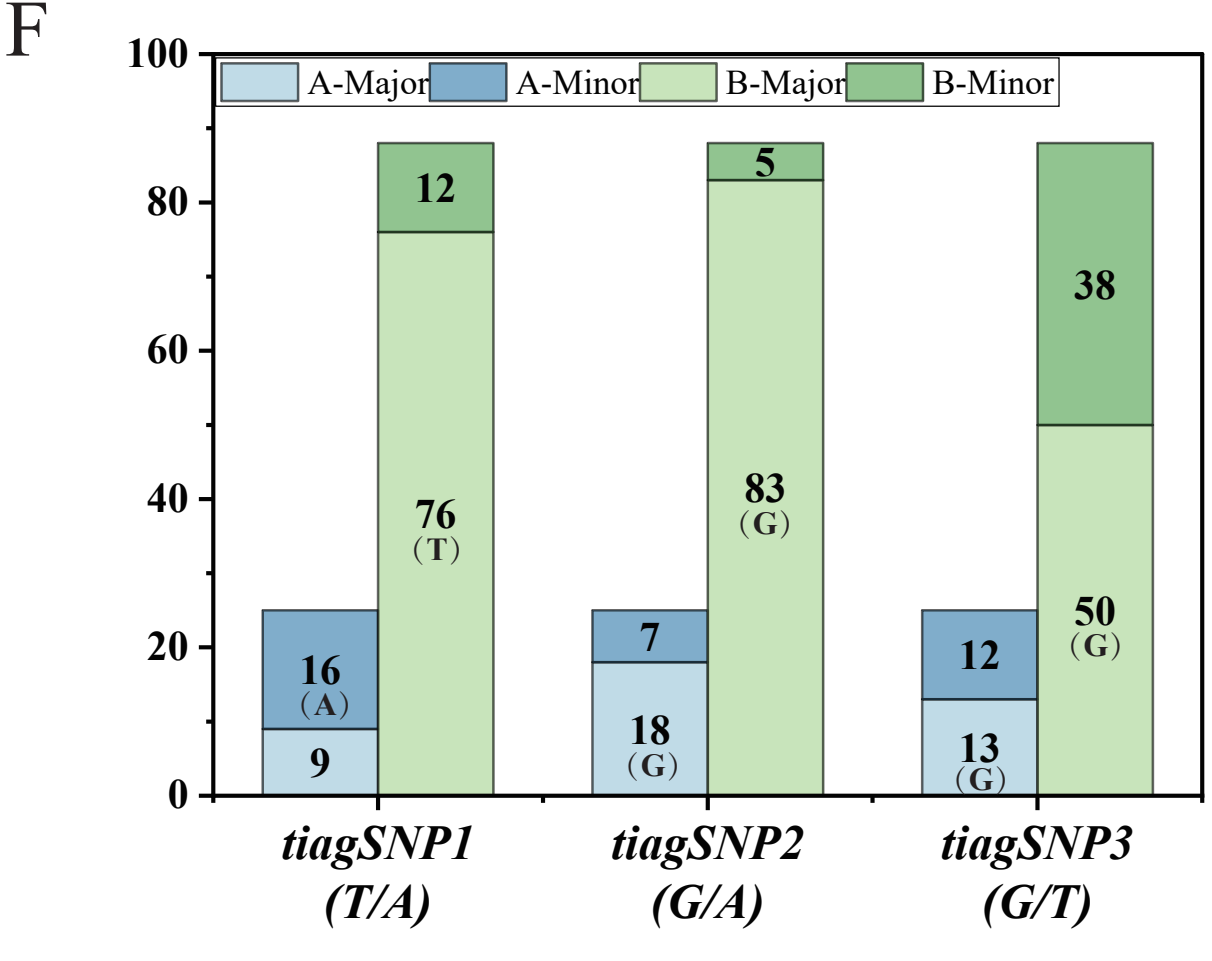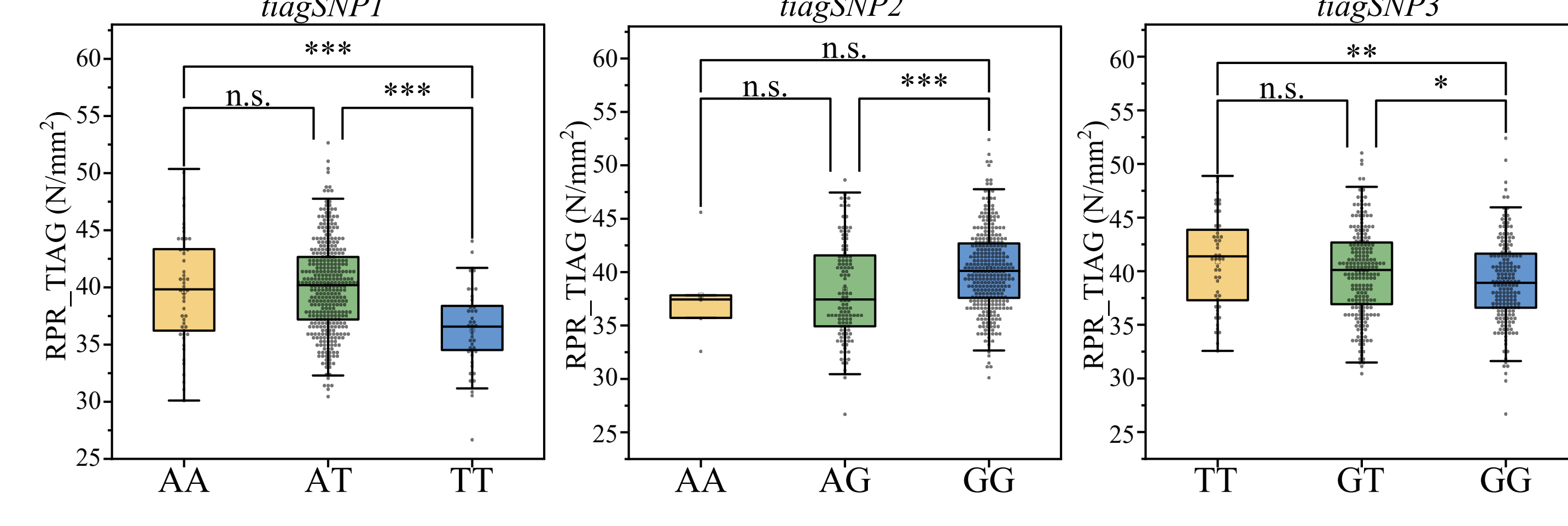

Supplement: Supplementary file 1 [file ijms-25-01190-s001.zip › ijms-2770949-supplementary/Supplementary figures/Figure S4. Allele frequency and genotypic value distribution of main QTNs for 6 traits.pdf]

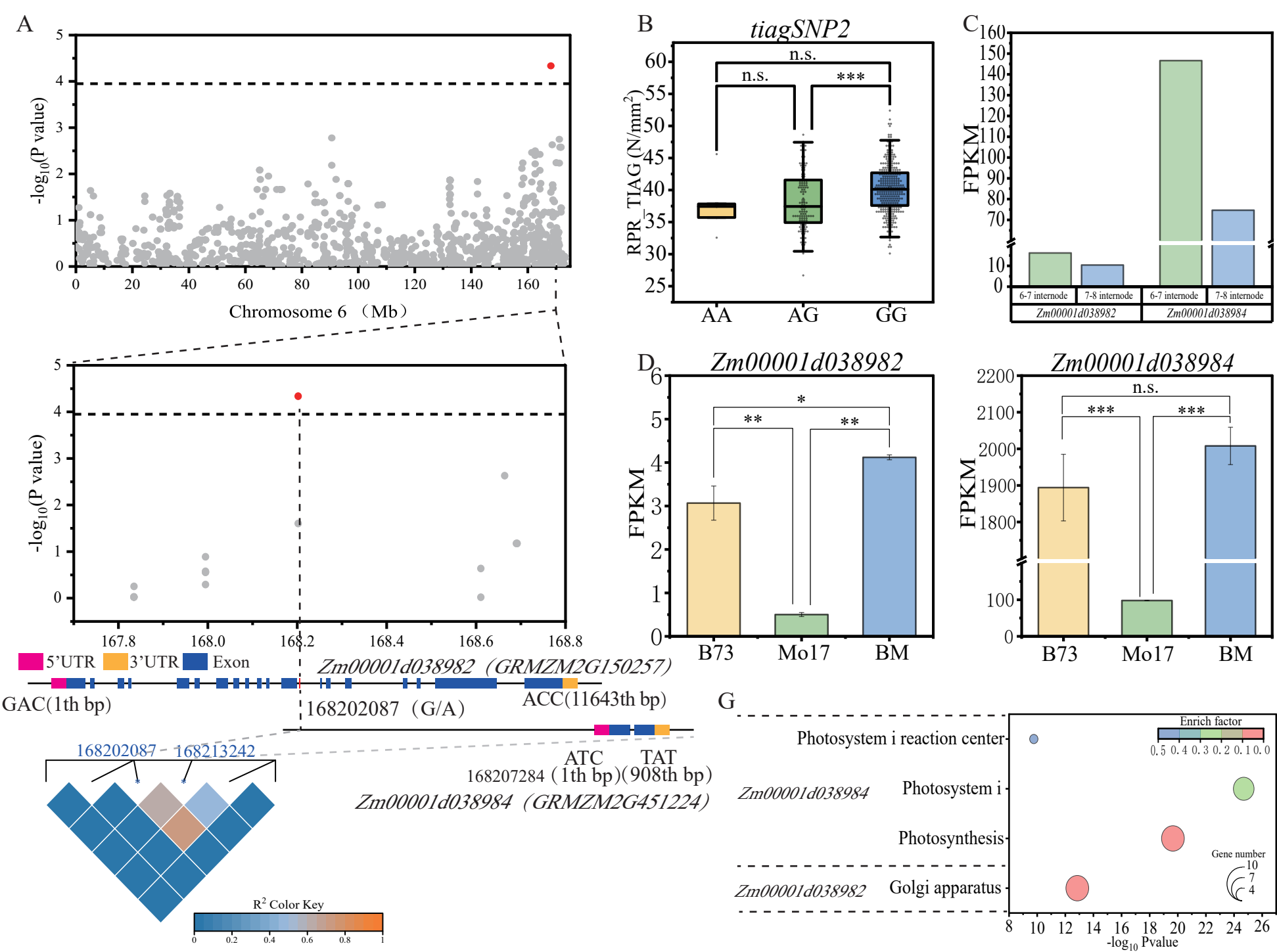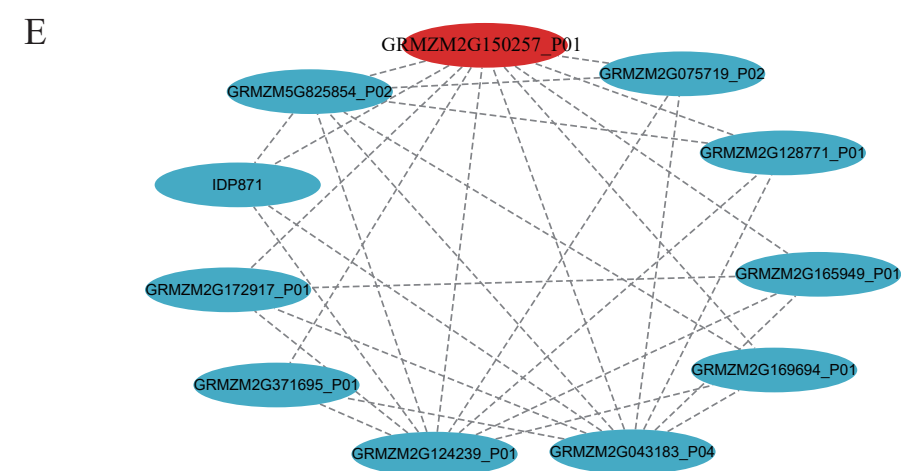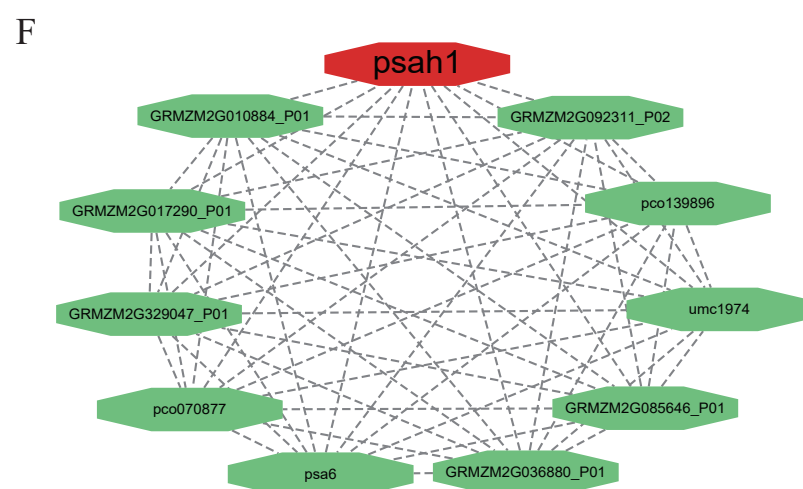

Supplement: Supplementary file 1 [file ijms-25-01190-s001.zip › ijms-2770949-supplementary/Supplementary figures/Figure S5. Multi-omic identification of candidate genes Zm00001d038982 and Zm00001d038984.pdf]

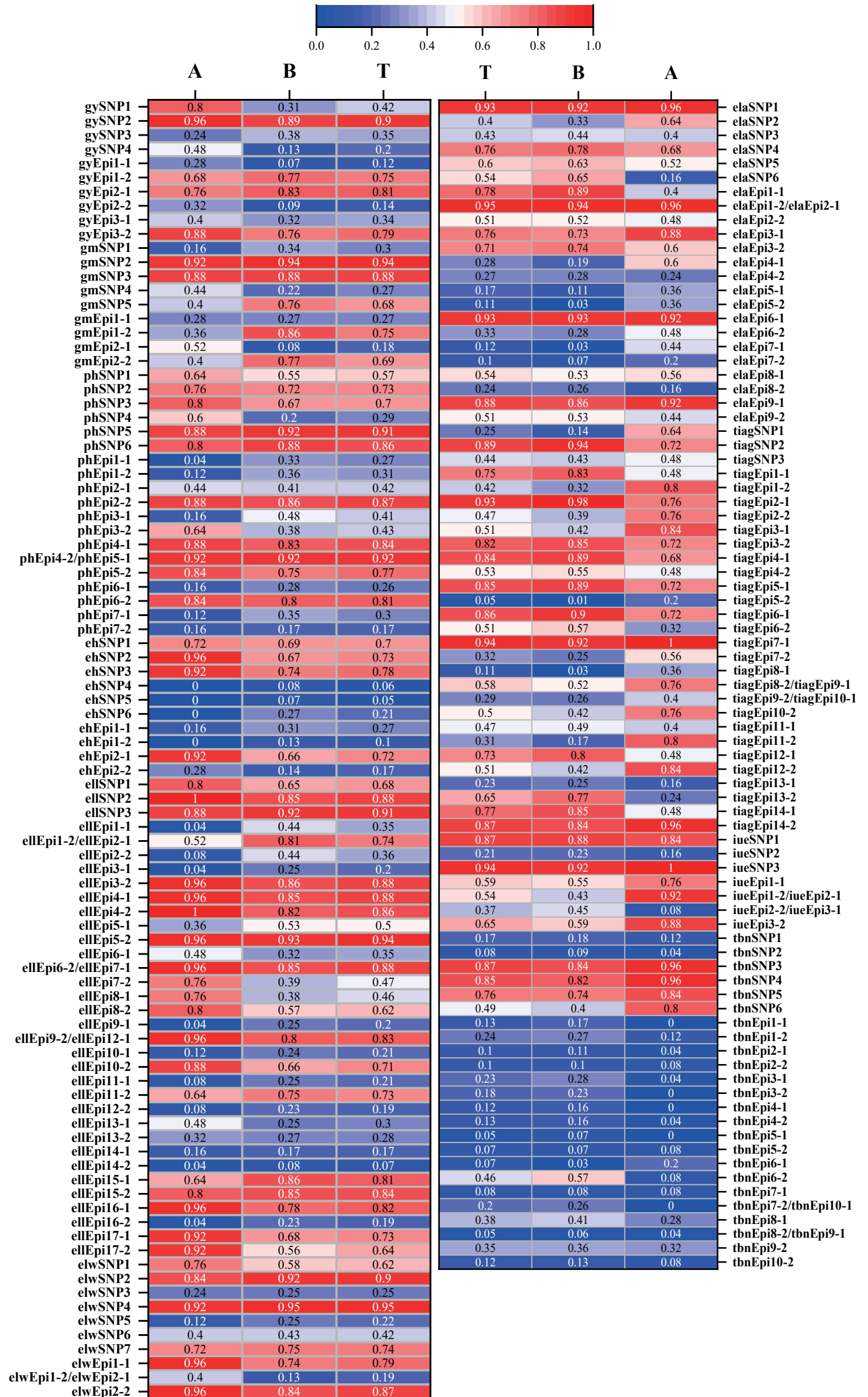

Supplement: Supplementary file 1 [file ijms-25-01190-s001.zip › ijms-2770949-supplementary/Supplementary figures/Figure S6. Favorable genotypes enrichment within ShaanA, ShaanB, and Total population.pdf]

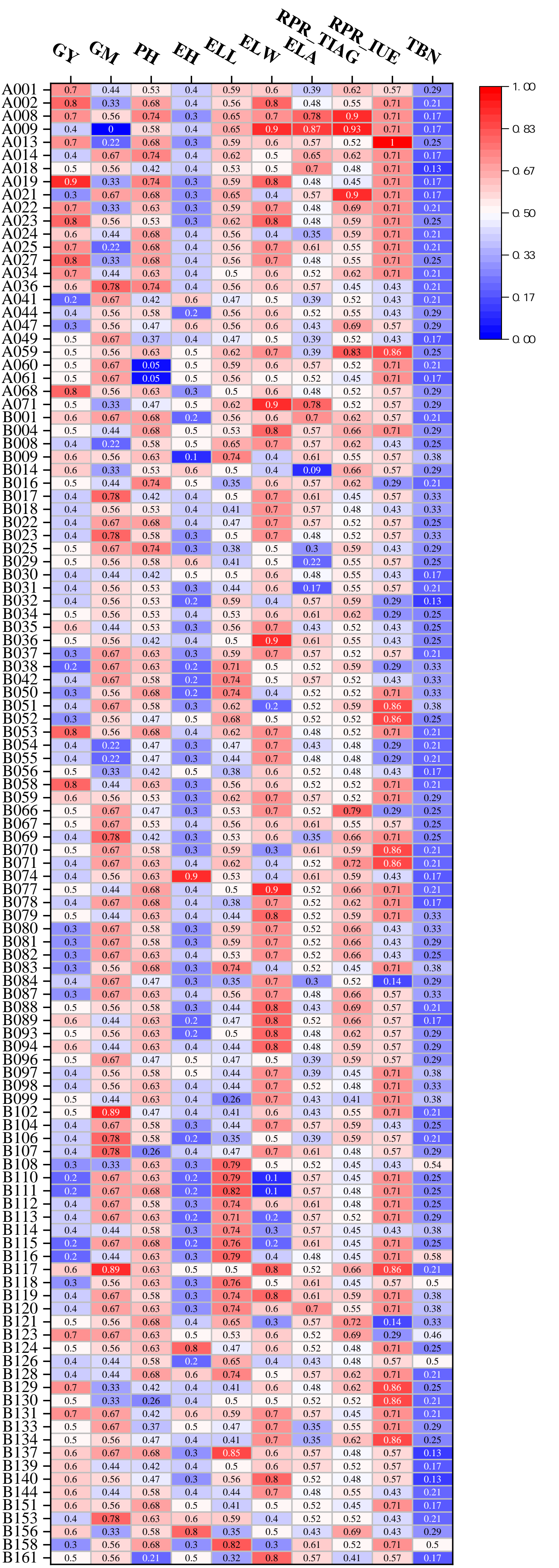

Supplement: Supplementary file 1 [file ijms-25-01190-s001.zip › ijms-2770949-supplementary/Supplementary figures/Figure S7. Favorable genotypes enrichment in each inbred line.pdf]
